# Costing of an Australian general practice COVID-19 drive-through testing and respiratory clinic

**DOI:** 10.1186/s12875-022-01664-4

**Published:** 2022-03-29

**Authors:** Patrick Abraham, Jo-Anne Manski-Nankervis, Ruby Biezen, Christine Mary Hallinan, Katherine B. Gibney, Lena Sanci, Jemimah Ride

**Affiliations:** 1 Health Economics Unit, Centre for Health Policy, Melbourne School of Population and Global Health, University of Melbourne, Melbourne, Australia; 2 Department of General Practice, Melbourne Medical School, University of Melbourne, Parkville, Australia; 3 Department of Infectious Diseases, The University of Melbourne at the Peter Doherty Institute for Infection and Immunity, Melbourne, Australia

## Abstract

**Background:**

Responding to the COVID-19 pandemic requires safe and efficient testing on a large scale over a prolonged period. Outpatient testing facilities can clinically assess and test symptomatic individuals and test asymptomatic contacts. This study identified the resources required to establish and maintain an Australian general practitioner (GP) led testing facility that combined a respiratory clinic for clinical assessment and testing with a drive-through testing facility.

**Methods:**

Data were taken from clinic administrative records to identify the number of patients tested over the period April-June 2020. An independent auditor’s report identified the resources used in establishing, running, and staffing both clinics for the same period. Analyses were performed using the minimum and maximum daily throughput to understand the effect of demand on price per sample collected.

**Results:**

The respiratory clinic tested an average of 19 patients per day, at an estimated cost of $340.04 AUD. This varied to $687.99 AUD during the lowest demand scenario, and $281.04 AUD during the high demand scenario. The drive-through clinic tested an average of 47 patients per day, at an estimated cost of $153.57 AUD. This varied to $279.51 AUD during the lowest demand scenario, and $99.92 AUD during the high demand scenario.

**Conclusion:**

This study provides insight into the cost of testing at a drive through and respiratory clinic in Australia. The evidence highlights importance of considering variation in demand and the impact on efficiency, particularly where resource use is fixed in the short term.

**Supplementary Information:**

The online version contains supplementary material available at 10.1186/s12875-022-01664-4.

## Introduction

The COVID-19 pandemic caused by the severe acute respiratory syndrome coronavirus 2 (SARS-CoV-2) virus was first detected in December 2019, and rapidly spread globally. Just over a year later, most countries are still implementing significant public policy responses to the pandemic. Large scale and rapid testing is a key element of managing the pandemic, to diagnose and manage cases and their close contacts [1]. Testing of those with symptoms, those who are close contacts of positive cases, and population screening in high risk areas are important public health measures to manage outbreaks and inform setting of restrictions [2]. Accurate and timely testing can facilitate return to work and other activities, and may shorten the duration of isolation and quarantine [3]. Adequacy of testing capacity therefore has substantial economic and social implications for communities during the pandemic.

There are three main components in a large-scale testing system: sample collection, laboratory processing of samples, and management of results. This paper focuses on sample collection, which occurs in several settings including hospital-based clinics, mobile vans, drive-through sites, airports, workplaces, and general practice clinics. Some of these settings only manage sample collection, however some patients also need clinical assessment at the time of testing.

In March 2020, the Australian Government Department of Health announced the funding of approximately 100 respiratory clinics across the country with the goal of providing care to acute respiratory patients while reducing the COVID-19 screening workload at general practices and hospitals so they could continue to provide care for non-COVID-19 related acute and chronic conditions [4]. These clinics triage, assess, and treat symptomatic patients and collect COVID-19 testing samples where appropriate according to local testing recommendations [5]. The clinic that is the focus of this study was established in Melbourne in April 2020, at a time when the state of Victoria, Australia, had 1190 confirmed cases of COVID-19. At the time vaccines had not yet been developed, and the Australian public health response relied on testing, contact tracing and other non-pharmaceutical interventions [6]. This clinic operated as a General Practitioner led drive-through testing clinic (DTTC) in parallel to the respiratory clinic (RC). The RC was for patients who required clinical assessment within the facility, whilst the DTTC was for patients who only required testing and thus could remain in their car [5, 7]. These two streams shared staff and some resources for triage and brief clinical assessment, as the appropriate clinic was determined for each patient.

A high-quality testing system requires significant resource allocation [8]. Sample collection needs to be conducted in accordance with infection control and clinical guidelines to decrease the risk of transmission of SARS-CoV-2 and ensure an adequate sample is collected [9]. Testing may be funded by government, public or private health insurance, patient out-of-pocket costs, or a combination of these. In Australia, sample collection mostly occurs in publicly-funded testing facilities with no out-of-pocket cost incurred by patients [10]. Apart from laboratory fees [11], there is no publicly available information on the input costs of testing (the resources needed for testing) in Australia. Internationally, the cost per patient with a mobile testing facility was found to be £55 in Scotland (equivalent to $100AUD) [12], and in the United States $164 USD ($235AUD) at a nurse-led drive through testing clinic, and between $315 and $514 USD ($454 to $740 AUD) at respiratory clinics [13]. Given the numbers of tests conducted daily throughout the pandemic (4,300–93,000 per day in Australia [14] and 100,000–2 million per day in the US [15]), and the broader economic implications of the pandemic, understanding the cost implications of the testing component is important.

This paper documents the resources required to establish and maintain a GP-led (General Practitioner) drive-through and respiratory clinic in Melbourne, Australia, addressing the costs of sample collection in this type of facility.

## Methods

### Context

The RC and DTTC were established during the first wave of COVID-19 in Australia. This study uses data from 3 April to 30 June 2020, during which time Australia had recently entered stage 3 of lockdown, which broadly consisted of stay at home directives (bar essential services), two person limits on gathering size, and strict social distancing measures [16]. During the observation period, case numbers reduced and Victorian restrictions were eased into stage 2 (broadly, relaxing of stay at home directives to include social gatherings, resumption of outdoor recreational activities and resuming all elective surgeries) [17]. The clinic was geographically located near a high intensity of locally acquired cases in the west of Melbourne, particularly during Melbourne’s second wave of the pandemic, from July through October. Most of the second wave of high case numbers occurred slightly after the observation period, although the clinic continued to operate through that period.

### Data source

This study was conducted alongside an observational safety study conducted at the clinic (manuscript in progress). Data were extracted from clinic financial records and electronic medical records (EMR). EMR data extracted using the GRHANITE tool [18] and stored in the University of Melbourne Data for Decisions Patron dataset [19] were used to identify the number of patients tested at the RC and DTTC per day. An independent auditor’s review provided costs of the resources required to run the clinics from April-June 2020. This study was reviewed and approved by the University of Melbourne Human Ethics Subcommittee (Ethics ID2056712 and ID2057310), and all methods were performed in accordance with the relevant guidelines and regulations from this committee. Written consent was obtained from participating general practice staff and a waiver of consent was approved to access de-identified patient data from the participating practice’s electronic medical records.

### Costing

Resource use was classified into four categories: fixed, running, staff, and personal protective equipment (PPE). PPE costs were not fully borne by the clinic, being partly supplied and/or subsidised by government, but these resources are included in the costing for completeness. Costs were attributed on a per-patient basis by attributing all resources consumed to the number of patients presenting for testing. Resources were shared between the RC and DTTC, so the attribution of resources needed to account for the numbers presenting to both clinics. During the period of April–May there were changes to both the numbers of patients presenting for testing (thought to be related to media reporting and calls for testing in response to outbreaks of COVID-19 in the community) and the resourcing of the clinic in terms of staffing. We therefore divided the period of observation into two phases of analysis: the start-up phase (April) and a maintenance phase (May–June).

Fixed costs cover one-off resource investment to establish the clinic. These were attributed to RC and DTTC item-by-item depending on their function in either clinic, with shared resources allocated with a 2:1 ratio between RC and DTTC respectively, upon recommendations from the clinic administrators. The duration of the clinic’s operation was estimated at 9 months by the clinic administrators, which in combination with the number of patients per day was used to determine the fixed cost per patient. Running costs including rental and cleaning fees and were attributed evenly between the two clinics and were independent of the number of patients seen in each clinic. PPE costs were assumed to depend on the number of patients attending the clinic and included gloves, gowns, goggles, visors, and masks. The analysis assumed PPE was used at a rate determined by the protocol (i.e., which staff members should wear gloves, masks, full PPE) and that one face mask was issued per patient. We also allowed for extra usage to account for unplanned changes of PPE and extra face masks issued to people in attendance in the cars with patients. Staff costs were based on the relevant hourly rate of pay and allocated to each clinic from the total number of rostered hours of each type of staff member divided by the number of patients per day being tested. From the observational data in the safety study, the time taken per patient for the GP and Clinical Health Assistant (CHA) conducting the testing in the respiratory clinic was estimated to be approximately 15 min, compared with 5 min in the drive-through clinic, so the time cost of these roles was allocated with a 3:1 ratio of their time divided between the RC and DTTC respectively. For staff other than GPs and CHAs, the time spent per patient was reported to be the same whether they attended the respiratory or drive through clinics, so for these roles the proportion of time costs attributed to each clinic was determined by the proportion of patients attending each clinic on average.

### Costing the variation in demand

Clinic management needed to plan capacity in terms of the demand the clinic would be able to meet, despite uncertainty over the demand that would actually be faced (numbers of patients presenting for testing and duration of the need for the clinic to be maintained). This was determined to be a maximum of 80 patients per day tested through both clinics combined. While some changes to resources could be made according to demand, such as adjusting up or down the number of staff rostered on, this was not able to be changed rapidly on a day-to-day basis, thus to some extent the cost was fixed irrespective of the number of patients actually tested. To account for this extrinsically determined variation, we assigned the full amount of staff time rostered at the clinic across the number of patients tested, whether or not this amount of time was actually used per patient. For example, for a nurse role in phone triage rostered 2 × 4-h shifts per day, if there were 80 patients attending per day then each patient is assigned 1/80^th^ of the cost of that role’s time, while if 40 patients attend per day then each patient is assigned 1/40^th^ of the cost. The efficiency of the clinic (the number of patients tested using a given amount of resources), therefore varied with throughput. Periods of lower efficiency are a trade-off with capacity to respond to higher demand. To investigate the scale of this trade-off, we conducted scenario analyses to understand how the change in demand impacted the cost per patient. Given the daily fluctuation in demand during the observation period, we ran analysis at both minimum (36) and maximum (80) patients per day seen during this period. The mean number of patients tested in both clinics per day over the observation period was 66. Clinic management stated that scaling up to 120 patients per day would require the addition of an extra GP. We present the main results based on the mean number of cases per day (66 patients per day), and three alternate scenarios – low observed demand (36 patients per day), high observed demand (80 patients per day), and additional capacity (120 patients per day with an additional GP).

## Results

**Fig. 1:**
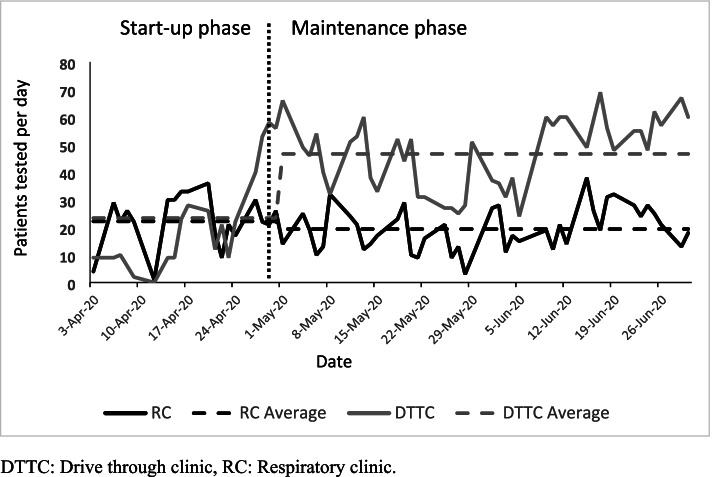
Patients seen per day in Respiratory and Drive through testing clinics *DTTC* Drive through clinic, *RC* Respiratory clinic

Daily patient throughput increased in the DTTC from the start-up (April) to maintenance phase (May–June) (23.4 vs 46.7) yet remained fairly consistent at the respiratory clinic (22.2 vs 19.4) (see Fig. 1). We use numbers from the maintenance phase as the base case in our analysis, as this represented a more consistent patient throughput.

### Patient demographics

During the observation period the total number of patients seen in the RC and DTTC was 1240 and 2418 respectively (total of start-up and maintenance phases). The demographic characteristics of patients attending the two clinics were similar in terms of age, sex, and whether they were a usual patient of the clinic (see Table 1).

**Table 1:** Respiratory Clinic and Drive Through Testing Clinic Patient Characteristics (*n* = 3658)

Trait	Categories	RC Number (%)	DTTC Number (%)
**Number of patients**		**1,240**	**2,418**
**Age in years**	**Mean**	**32.5**	**36.4**
**Gender**	**Female**	**737 (59.4)**	**1,455 (60.2)**
	**Male**	**501 (40.40)**	**963 (39.83)**
	**Missing**	**2 (0.16)**	**0**
**Usually attended the clinic**	**Usual patient**	**718 (57.90)**	**1,419 (58.68)**
	**New patient**	**522 (42.10)**	**999 (41.32)**
**Pathology results**	**SARS-CoV-2 detected**	**1 (0.08)**	**2 (0.08)**
	**Other pathology detected^**	**25 (2.02)**	**8 (0.33)**

^Other tests (such as influenza or herpes) were sometimes performed

### Cost per patient

**Fig. 2:**
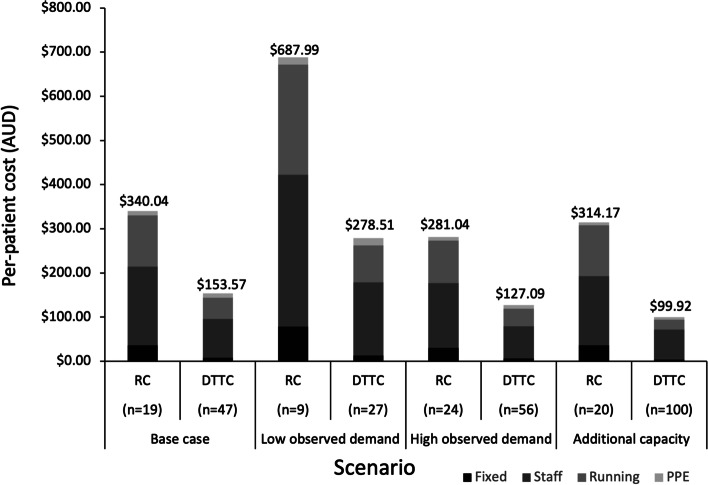
Cost per patient seen at Respiratory and Drive-through clinics in the base case and scenario analyses

In the base case analysis, the RC tested 19 patients per day, with an average cost per patient of $340.04, which comprised 11% fixed costs, 52% staff costs, 34% running costs and 3% PPE. The average cost per patient in the DTTC with 47 patients tested per day was $153.57, comprised of 5% fixed costs, 57% staff costs, 31% running costs and 6% PPE costs (see Fig. 2).

Under the low observed demand scenario, the average cost per patient in the RC with 9 patients per day was $687.99, and in the DTTC with 27 patients per day was $278.51. The high demand scenario (the maximum number seen during the observed period, with no staffing increase) had an average cost per patient in the RC with 24 patients per day of $281.04, and the DTTC had an average cost per patient of $127.09 with 56 patients per day. Finally, in the additional capacity scenario with an extra GP, the average cost per patient was $314.17 in the RC and $99.92 in the DTTC. Observed results and scenario analysis results are displayed in Fig. 2.

## Discussion

This study adds to a scarce literature on the costs of testing during the COVID-19 pandemic. It demonstrates the resources needed to implement this novel model for sample collection, combining a GP-led drive-through clinic with a GP respiratory clinic. It also demonstrates the important tension between efficiency and responsiveness (having capacity to respond to changing demand for testing over the course of the pandemic). Cost per patient over a feasible range of demand scenarios ranged from $278 to $688 in the RC, and $100 to $281 in the DTTC. These are similar to the costing estimates available in the US, with approximately $200 AUD for a drive-through test, and $500 AUD for a respiratory clinic visit per patient [13]. Conversely, the Scottish study found a cost of approximately $100 AUD [12], this being lower than our findings, however was also not a drive-through testing site. The comparative cost of other models of testing in Australia is unknown, with no publicly available data on testing in standalone drive-through testing facilities, hospital clinics, or GP respiratory clinics without the drive-through component in Australia. While these findings and unit costs are specific to an Australian context, the lack of data globally indicates the cost of sample collection elsewhere may not be frequently analysed. Given the substantial investment and importance of testing globally, the cost of sample collection from this study highlights the importance of analysing the economic cost of testing clinics elsewhere.

This clinic has been elsewhere shown to meet high infection control standards (manuscript in progress) and keeps patients inside their cars where possible, to reduce the risk of disease transmission from patient to health workers [8]. Additionally, combining the drive through component with a general practice model allows for patients with more severe symptoms to have a thorough medical assessment along with their sample collection within the same facility, partly reflected in the other pathology detected in laboratory results at this clinic. The benefits of this medical assessment are not captured by the metric of number of patients tested, hence the lower cost per test in the DTTC should not be interpreted as indicating greater efficiency given the difference in purpose and clinical needs of patients attending each clinic. To determine the true value of these additional components would require an evaluation comparing this type of clinic to another testing structure and assessing both costs and patient health outcomes.

Our analysis has highlighted the trade-off between responsiveness and efficiency in such a testing facility. After the observation period for this study, Melbourne went through a period higher case numbers with increased demand for testing, followed by a strict lockdown period and then a period without community transmission [20]. Throughout there were large fluctuations in demand for testing, which would have impacted the efficiency of the clinic. In the low demand scenario we described, the cost per patient was more than double that in the base case. Policy makers and funders need to understand the trade-off inherently built into testing structures, between running efficiently and responding to demand fluctuation. It is beyond the scope of this paper to determine what is the optimum balance between the two, but these results provide information to decision makers seeking to understand this trade-off.

### Limitations

The major limitation of this study was that it was conducted at a single site. Without comparison to another facility, we cannot assess the generalisability of the results to other testing facilities. This study would benefit from a cost comparison to another testing structure such as a hospital testing facility, another GP respiratory clinic, or a drive-through testing site without attached GP respiratory clinic component, to understand the differences in resource usage between structures. It is understood that combining the respiratory component with the DTTC is a novel structure, hence we would advise caution in interpreting these results in the context of other testing sites. By excluding the patient numbers for the first month of clinic operation, we have avoided underestimating the average throughput for the DTTC, providing a more representative estimate to determine the average cost per patient. However, we do not have the data from the highest peak of demand during Melbourne’s second wave that occurred just after the observation period, and so our range of estimates may under-estimate the peak efficiency this model may have achieved. Staff costs were the largest component of costs in both the DTTC and RC. This analysis used the hourly rates paid at this clinic; however, these may not apply at other clinics in the absence of standard rates of pay for the COVID testing clinic workforce in Australia, and for GPs in general in Australia. With high demand for skilled staff willing to work in such clinics with the associated risks and burden, rates of pay in testing clinics may exceed usual rates of pay for all staff members. Other clinics may use a different mix of staff roles. There were also costs associated with the space required to accommodate waiting areas as well as the RC and DTTC themselves, which may differ in other clinics. Despite these limitations, this study provides valuable information for decision makers in the absence of any other published cost data in Australia and very little internationally.

## Conclusion

While the COVID-19 pandemic has moved into new phases with the introduction of vaccines, new variants, and the lateral flow testing, sample collection for polymerase chain reaction testing continues to be an important element of the public health management of pandemic response. While safety and infection control are vital to these testing structures, this study has shown that there are substantial economic implications of sample collection and patient care, including finite staff resources, which need to be explicitly considered in health system planning.

## Supplementary Information

**Additional file 1.**

## Data Availability

The datasets generated and/or analysed during the current study are not publicly available due to clinic confidentiality, but are available from the corresponding author on reasonable request.

## Abbreviations

AUD: Australian dollar
CHA: Clinical health assistant
DTTC: Drive-through testing clinic
EMR: Electronic medical record
GP: General practitioner
PPE: Personal protecting equipment
RC: Respiratory clinic
SARS-CoV-2: Severe acute respiratory syndrome Coronavirus 2
