# Supplementary material for: Costing of an Australian general practice COVID-19 drive-through testing and respiratory clinic
Source: BMC Prim Care. 2022 Mar 29;23:58. doi: 10.1186/s12875-022-01664-4 (PMC8962929; doi:10.1186/s12875-022-01664-4)
Supplement: Supplementary file 1 — Additional file 1. [file 12875_2022_1664_MOESM1_ESM.docx]

Supplementary material

**Supplementary Table 1: Breakdown of costs (AUD) in the base case and scenario analyses**

|  | Base case | | Low observed demand | | High observed demand | | Additional capacity | |
| --- | --- | --- | --- | --- | --- | --- | --- | --- |
|  | **RC** | **DTTC** | **RC** | **DTTC** | **RC** | **DTTC** | **RC** | **DTTC** |
| **Fixed** | 36.37 | 7.57 | 78.46 | 13.08 | 30.03 | 6.25 | 36.37 | 3.53 |
| **Staff** | 178.23 | 88.13 | 343.50 | 165.87 | 147.15 | 72.76 | 155.92 | 67.79 |
| **Running** | 115.76 | 48.19 | 249.71 | 83.24 | 95.59 | 39.79 | 115.76 | 22.47 |
| **PPE** | 9.68 | 9.68 | 16.33 | 16.33 | 8.30 | 8.30 | 6.13 | 6.13 |
| **TOTAL** | 340.04 | 153.57 | 687.99 | 278.51 | 281.04 | 127.09 | 314.17 | 99.92 |

**Supplementary Table 2: Components of resources**

| Fixed costs | Running costs | Staff | PPE |
| --- | --- | --- | --- |
| Medical Supplies  Communications (telephones, website updates)  Infrastructure (temporary buildings, equipment, meeting room hire for training, infectious waste bins)  Personnel (computer consultancy, legal fees, development costs, administration contract) | Laundry  Accounting  Electricity  Room hire  Viral swabs  Security  Stationery and printing  Software  Car park rental  Hand sanitiser  Staff amenities  Payroll administration fee  WorkCover | Nurse (phone triage)  GP (overseer) CHA (result processing)  Receptionist  Scribe Steward (holding bay)  Steward (side gate) Nurse (clinic)  GPs (clinic)  CHAs (clinic) | Masks  Gloves  Visors  Goggles  Gowns  Pathology bags  Swabs |

GP: General Practitioner, CHA: Clinical Health Assistant
